# Failure of ProTaper rotary Ni-Ti instruments used by undergraduate students

**DOI:** 10.4317/jced.50735

**Published:** 2012-10-01

**Authors:** Marc García-Font, Fernando Duran-Sindreu, Sergio Morello-Castro, Montserrat Mercade-Bellido, Rufino Bueno-Martínez, Miguel Roig-Cayón

**Affiliations:** 1 DDS. Professor of Endodontics. Department of Restorative Dentistry and Endodontics, Faculty of Dentistry, Universitat Internacional de Catalunya, Barcelona, Spain.; 2 DDS, PhD. Professor of Endodontics. Department of Restorative Dentistry and Endodontics, Faculty of Dentistry, Universitat Internacional de Catalunya, Barcelona, Spain.; 3 DDS, PhD. Professor of Endodontics and Head of Department of Restorative Dentistry and Endodontics, Faculty of Dentistry, Universitat Internacional de Catalunya, Barcelona, Spain.

## Abstract

Objective: To evaluate the effect of number of uses, angle and radius of curvature and type of instrument on the fracture of ProTaper rotary instruments when used by undergraduate students. Study Design: Three hundred and seventy-six molars, with a total of 1114 root canals, extracted were instrumented by undergraduate students using ProTaper instruments according to the manufacturer´s recommendations. When fracture occurred, data were collected concerning the number of uses, type of instrument, level of fracture, angle and radius of curvature. ANOVA test were used to determine the influence of type of instrument in the incidence of instrument fracture. Logistic regression model was used to determine the influence of number of uses, angle and radius of curvature in the incidence of instrument fracture. Significance was set at p< 0.05. Results: A total of 37 Ni-Ti rotary instruments fractured during the treatment. Fracture occurred in 9.84% (37/376) of the teeth treated and 3.32% of the canals prepared with Ni-Ti rotary instruments (37/1114). A decrease in the radius of curvature of the canal significantly increased the likelihood of fracture (p=0.0001). Instrument fracture significantly increased as the number of uses increased (p=0.0037). No significant differences were found between the 6 types of ProTaper instruments (p=0.8). A reduction in the angle of curvature did not produce a significant decrease in the incidence of instrument separation (p=0.08). Conclusions: The results of this study imply that instrument fracture is linked to radius of curvature and number of uses.

** Key words:**Fracture, ProTaper ®, root canal preparation, undergraduate students.

## Introduction

Canal shaping is a critical part of endodontic treatment because it influences the outcome of the treatment (1).

The advent of nickel-titanium (Ni-Ti) rotary instrumentation has revolutionized root canal treatment by reducing operator fatigue, preparation time, and procedural errors associated with root canal instrumentation (2). However, there is a potential risk of rotary Ni-Ti instruments fracturing within the canals (3).

Fracture of instruments used in rotary motion may occur either by torsion or by cyclic fatigue. Torsional fracture occurs when an instrument tip or another part of the instrument is locked in a root canal while the shank continues rotating. When the torque exerted by the handpiece exceeds the elastic limit of the metal, tip fracture becomes inevitable (4).

The phenomenon of repeated cyclic fatigue may be an important factor in instrument separation. When instruments are placed in curved canals, they deform and undergo stress. Half of the instrument shaft on the outside of the curve is in tension, whereas the half on the inside is in compression (5). Consequently, each rotation causes the instrument to undergo one complete tension-compression cycle. Various factors have been associated with the fracture of Ni-Ti rotary instruments: number of uses, rotational speed, angle and radius of curvature (6), instrument design, instrumentation technique, torque, excessive apical force during instrumentation (7) and operator experience (8). The influence of operator experience has been assessed in several studies that showed training or experience was necessary to minimize the incidence of instrument separation (8,9). Mandel *et al.* (9) assessed the effect of the operator on ProFile® rotary Ni-Ti instrument fracture. The results suggested that more instruments failed during the ‘learning period’ than during the “application period”.

ProTaper® Ni-Ti instruments (Dentsply Maillefer, Ballaigues, Switzerland) have been introduced with unique designs of variable tapers, convex triangular cross-sections, and non-cutting tips. According to the manufacturer, a progressively tapered instrument system reduces instrument fatigue and breakage potential. It has been re-ported the advantages of rotary preparation with Ni-Ti instruments over hand preparation. But the risk of instrument fracture is the main problem inherent in rotary instrumentation by inexperienced operators (10). However, the factors influencing the fracture of ProTaper® rotary instruments in root canal treatment performed by inexperienced students have not been widely studied. To our knowledge no study has been published on ProTaper® files fracture in undergraduate operators.

This study aimed to evaluate the capability of third-year dental students with no endodontic experience to use the ProTaper® System (Dentsply Maillefer) on extracted teeth, in order to determine the incidence of instrument fracture based on: number of uses, type of instrument, and angle and radius of curvature.

## Material and Methods

One hundred and fifty-eight maxillary molars and 218 mandibular molars, with a total of 1114 root canals were used in this study (4 molars per student). Those molars whose apices were not completely closed, those that had root resorption, root fracture or extensive caries were excluded.

Root canal treatments were performed by 94 third year dental students with no experience in endodontics. The students received eight hours of theory on Ni-Ti and stainless-steel files, instructions of use (K-files, ProTaper instruments), cyclic fatigue and torsional fracture causes and how to minimize instrument fracture.

Ninety-four of new ProTaper® NiTi rotary instruments, each containing SX, S1, S2, F1, F2 and F3 files, were used. Each operator received 1 set. The students were told to follow the manufacturer´s instructions and to consult their teachers if they had any doubts (6 students per teacher).

The apical third of the root was embedded in wax while the root was encased in a mixture of plaster and saw-dust. After the mixture had set, it was cut out in the form of a block and two radiographs (Mesio-distal and bucco-lingual) were taken.

Standard access opening was made using round burs and Endo-Z burs (Dentsply/Maillefer, Ballaigues, Switzer-land). The entrance to each canal was located with an endodontic explorer and a size 10 K-file was placed into the canal to verify canal patency. A size 15 K-file was placed into the root canal to determine the working length (WL). A customized jig was designed with silicone (Optosil P Plus® HERAEUS KULZER, Hanau, Germany) that provided a reproducible position for the digital dental X-ray sensor and for alignment of the cone. Two digital radiographs (Mesio-distal and bucco-lingual) were taken with Kodak Dental Digital 6100 and the WL was established 1 mm short of the radiographic apex.

**Figure 1:**
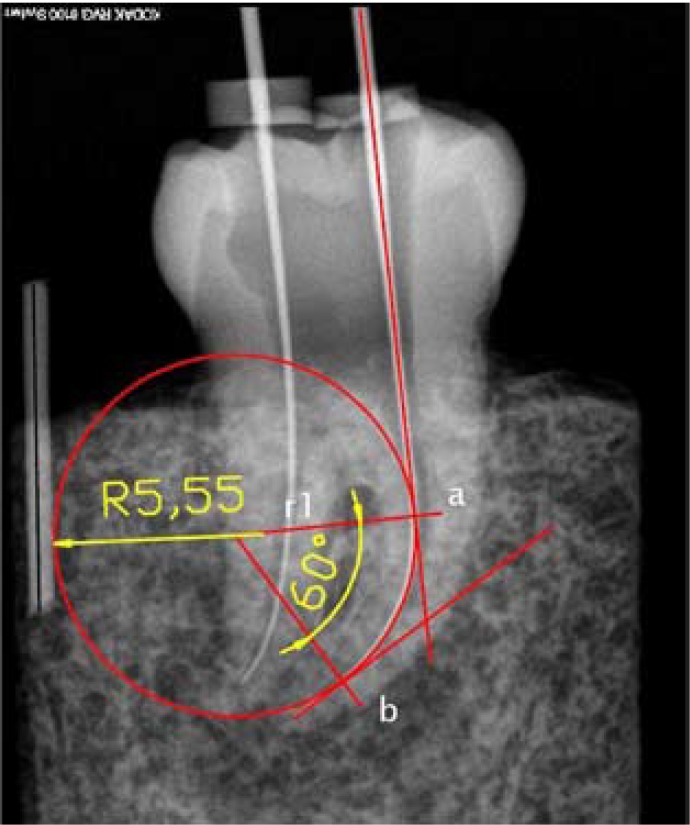
Determination of the angle and radius of curvature of the root canal. Method of Jonh Pruett et al. (6): (a) start and the (b) end of the canal curvature. (r1) radius of the curvature.

Radiographs were transferred to AutoCad 2008 (Autodesk Inc, San Rafael, CA USA) and the angle and radius of curvature of each root canal was determined following the methodology of Pruett *et al.* (6) (Fig. 1).

Instrumentation sequence

The root canal that had already been enlarged to a size 20 K-file was progressively instrumented with ProTaper® instruments. S1 was advanced to resistance but no more than two thirds of the canal depth. Then, the SX file was advanced to resistance to move the coronal aspect of the canal away to improve radicular access. This was followed by using S1 and then S2 to working length. The other files were used in the following sequence, and all were advanced to working length: S2, F1, F2, and F3, but the F3 did not always reach the working length. All instruments were used in an endodontic electric motor (Tecnika digital motor ATR, Pistoia, Italy) Following the manufacturer’s instructions.

During mechanical instrumentation root canals were irrigated with 4% NaOCl The instruments were wiped clean after each use, and then examined. Instruments that exhibited deformations were discarded and replaced with new ones.

When an instrument fracture occurred a radiograph was taken to confirm the fracture and the following information was recorded: type of ProTaper® instrument, level of separation and number of uses before failure. The instrument was replaced when the fractures occurred.

ANOVA test were used to determine the influence of type of instrument in the incidence of instrument fracture. Chi-square test was used to compare the incidence of instrument fracture for each root canal third. Logistic regression model was used to determine the influence of number of uses, angle and radius of curvature in the incidence of instrument fracture. Significance was set at p < 0.05.

## Results

Incidence of instrument separation

A total of 37 Ni-Ti rotary instruments fractured during the treatment. Fracture occurred in 9.84% (37/376) of the teeth treated and 3.32% of the canals prepared with Ni-Ti rotary instruments (37/1114).

Type of instrument

The percentages of instrument fracture according to type of instrument are shown in  Table 1. No significant differences were found between the 6 types of ProTaper® instruments (p=0.8) in the incidence of instrument fracture.

**Table 1:**
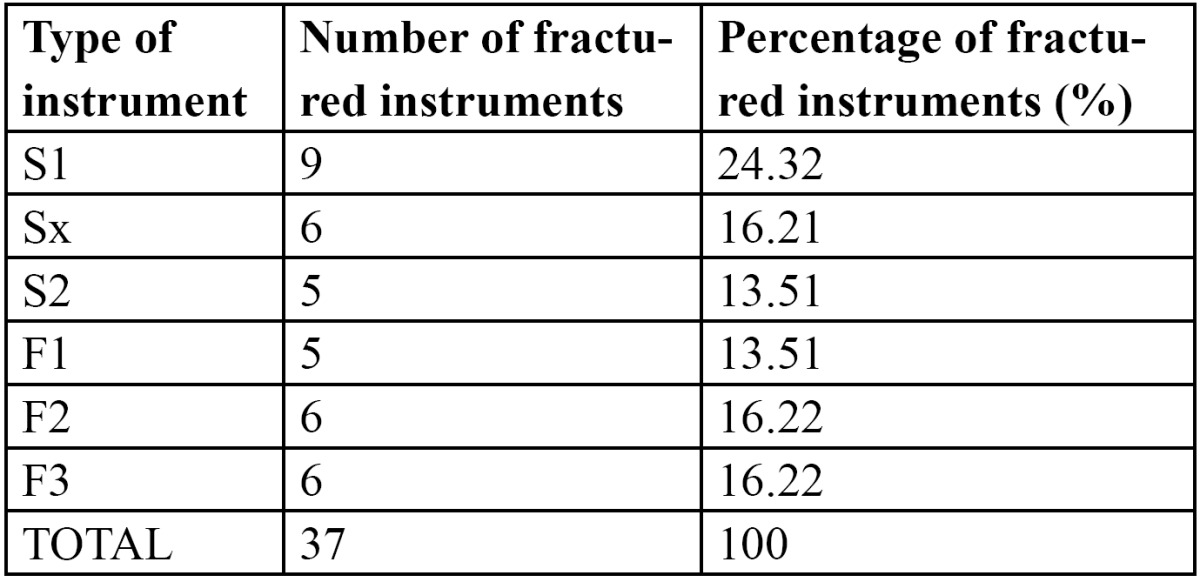
Separation for type of instrument.

Number of uses

In the current study, the authors tracked the number of teeth instrumented, instead of number of canals. Instrument fracture significantly increased as the number of uses increased (p=0.0037) ( Table 2). In the present study was observed a marked increase in instrument fracture after shaping 2 molars.

**Table 2:**
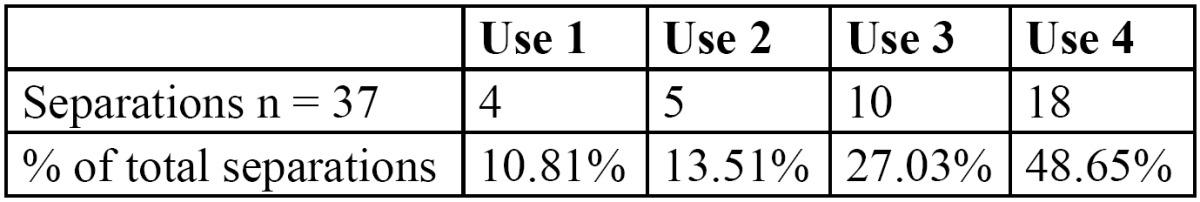
Fracture frequency according to number of uses of ProTaper® instruments.

Angle of curvature

The percentages and probability of instrument fracture according to angle of curvature are shown in  Table 3. A reduction in the angle of curvature did not produce a significant decrease in the incidence of instrument separation (p=0.08).

**Table 3:**
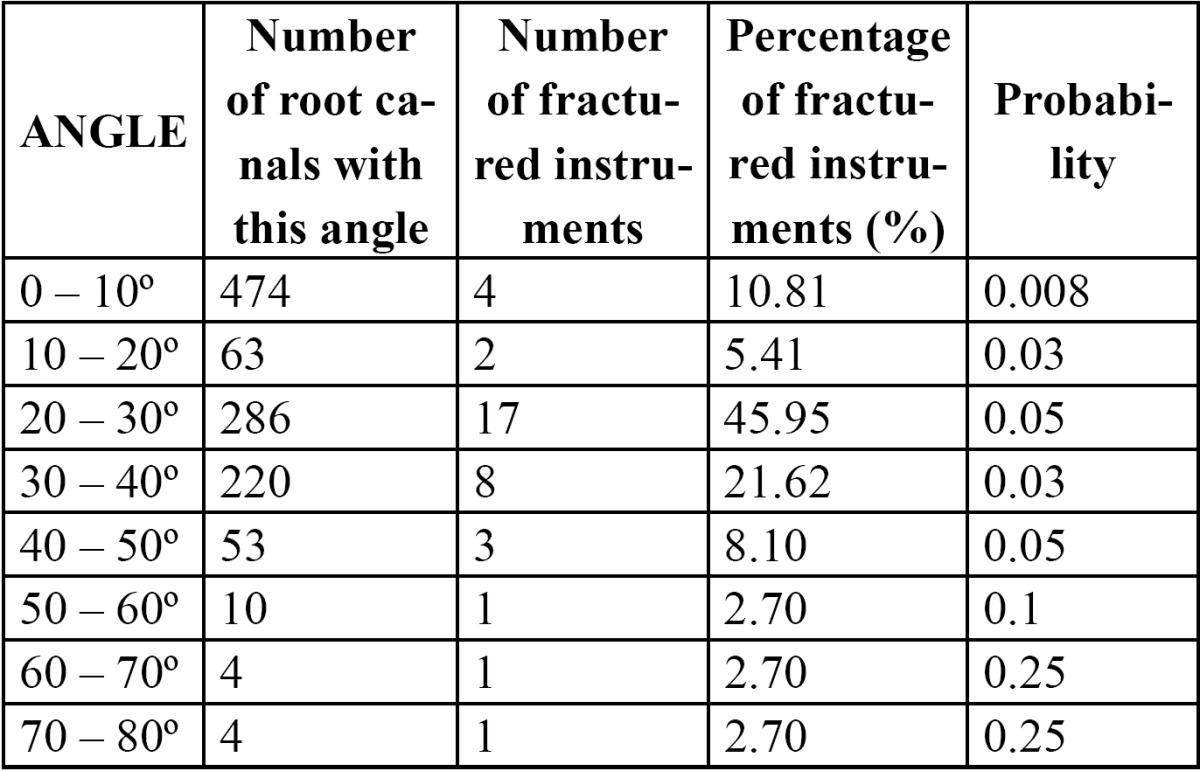
Percentage and probability of instrument fracture according angle of curvature.

Radius of curvature

Instrument fracture significantly increased as the radius of curvature decreased (p=0.0001) (Table 4).

Level of fracture

Of the 37 instruments fractured, 31 (83.78%) fractured in the apical third, 2 (5.40%) in the middle third, and 4 (10.81%) in the coronal third of the canals. Chi-square test revealed instrument fracture was more likely to occur in the apical third than in the coronal and middle third (p=0.0001).

## Discussion

Many authors have reported the advantages of rotary preparation with Ni-Ti instruments over hand preparation, for both experienced and inexperienced operators. But the risk of instrument fracture is the main problem inherent in rotary instrumentation by inexperienced operators (10). In this study extracted teeth were used because simulated canals in resin blocks do not reproduce the action of the instruments in root canals, due to differences in the surface texture, hardness and cross-sectioning. Glide path was created up to 20 K-file to WL before using ProTaper® instruments since manual pre-flaring of the root canal has been reported to increase the number of uses of the S1 instrument before failure, since manual pre-flaring drastically reduced torsional stress as the canal width becomes at least equal to the diameter of the instrument tip used (11).

In this study, there were a total of 37 (9.84%) fractured instruments from 376 molars instrumented by students with no experience in rotary instrumentation. These findings differed to those obtained by Di Fiore et al. (12). Di Fiore *et al.* (12) reported the incidence of rotary file fractures in molars instrumented by endodontic residents was 2.74%. This difference could be due to the fact that operator experience is an important factor when evalu-ating the frequency of fracture (9). Mandel et al. (9) observed that when other factors such as canal geometry, instrument speed, and sequence were maintained as constants, operator skill seemed to be an important cause of instrument fracture. These authors (8,9,13) found that the effect of operator experience was the most consistent and predictable parameter in instrument fracture. These findings are supported by Parashos et al. (13), who reported that the operator was the most important influence on defect rates of Ni-Ti instruments.

Wolcott et al. (5) observed that the F3 file demonstrated the highest frequency of fractures. These results may be explained by Grande et al. (14) and Inan *et al.* (15), who observed that cycles to failure decreased as the instrument volume increased. In the present study, however, the S1 demonstrated the most fractures, with 9 of 37 (24.32%). These differences could be put down to the fact that Wolcott *et al.* (5) enlarged coronal third canals with Gates Glidden and SX ProTaper® instruments before using the S1 instrument. But the present study corroborates Peng *et al.* (16), who observed that 38% of all discarded files were S1 instruments. The S1 instrument is used two times, instead of the single use for other instruments of the ProTaper® system. Logically, it is more likely to suffer from wear or damage; indeed, the manufacturer suggests that this instrument should be replaced more frequently.

In this study, canal curvatures were classified according to the method described by Pruett (6). This method was used because it has been widely applied and cited as a standard for classifying root canal curvatures in numerous studies (17,18). The fatigue of an instrument may be related to the degree of flexure it undergoes when placed in a curved root canal (17). The present research shows instrument fracture significantly increased as the radius of curvature decreased. These findings concur with those obtained by several authors (6,17) who observed that fatigue life of Ni-Ti rotary instruments was significantly influenced by the radius of curvature. Zelada *et al.* (17) reported that radius of curvature was the most important factor in instrument fracture, and in canals with a small radius of curvature the risk of instrument fracture was greater. A reduction in the radius of curvature similarly reduces the instrument’s ability to resist torsional forces (19). Hence, it was not surprising to find that most of the instruments in the present study fractured in the apical third where canals usually curve (20). These findings corroborate Iqbal *et al.* (20) who observed that the probability of instrument fracture in the apical third was more likely than in the coronal third and middle third.

The angle of curvature, however, was not a factor that influenced instrument fracture significantly, a fact, which contradicts the findings of other studies in which the angle was found to be significant (6,18). The discrepancy might be explained by the different methodologies used in the different studies (6,17,18).

In this research study, instrument fracture significantly increased as the number of uses increased. Reduction in the remaining fatigue life of used instruments is a common characteristic of the rotary Ni-Ti endodontic files, as previously reported by several authors for a number of instrument types (18,21). Patiño et al. (18) observed that the number of uses was the variable that was most significantly correlated with instrument fracture. Consequently, the discarding of the Ni-Ti instruments after a certain number of clinical uses is recommended (20). However, there is no consensus in the literature concerning a recommended number of uses of rotary Ni-Ti instruments, which varies from 1 to 27 canals, with a mean of approximately 11 canals (21). In the present study was observed a marked increase in instrument fracture after shaping 2 molars. This suggests that although new instruments can fracture at their first canal use, those that are used for 3 or more molars may have a higher probability for fracture. Therefore, it can be concluded from these differing findings that the number of uses of rotary Ni-Ti instruments will depend on a number of variables including instrument properties, canal morphol-ogy, and operator skill (13).

## Conclusions

The results of this study imply that instrument fracture is linked to radius of curvature and number of uses.

## References

[1] Pettiette MT, Delano EO, Trope M (2001). Evaluation of success rate of endodontic treatment performed by students with stainless-steel K-files and nickel-titanium hand files. JEndod.

[2] Iqbal MK, Firic S, Tulcan J, Karabucak B, Kim S (2004). Comparison of apical transportation between ProFile and ProTaper NiTi rotary instruments. Int Endod J.

[3] Ruddle CJ (2004). Nonsurgical retreatment. J Endod.

[4] Plotino G, Grande NM, Cordaro M, Testarelli L, Gambarini G (2009). A review of cyclic fatigue testing of nickel-titanium rotary instruments. J Endod.

[5] Wolcott S, Wolcott J, Ishley D, Kennedy W, Johnson S, Minnich S et al (2006). Separation incidence of Protaper rotary instruments: A large cohort clinical evaluation. J Endod.

[6] Pruett JP, Clement DJ, Carnes DL Jr (1997). Cyclic fatigue of nickel-titanium endodontic instruments. J Endod.

[7] Gambarini G (2001). Cyclic fatigue of nickel-titanium rotary instruments after clinical use with low- and high-torque endodontic motors. J Endod.

[8] Yared GM, Bou Dagher FE, Machtou P (2001). Influence of rotational speed, torque and operator’s proficiency on ProFile failures. Int Endod J.

[9] Mandel E, Adib-Yazdi M, Benhamou LM, Lachkar T, Mesgouez C, Sobel M (1999). Rotary Ni-Ti profile systems for preparing curved canals in resin blocks: influence of operator oninstrument breakage. Int Endod J.

[10] Sonntag D, Guntermann A, Kim SK, Stachniss V (2003). Root canal shaping with manual and rotary Ni-Ti files performed by students. Int Endod J.

[11] Berutti E, Negro AR, Lendini M, Pasqualini D (2004). Influence of manual preflaring and torque on the failure rate of ProTaper rotary instruments. J Endod.

[12] Di Fiore PM, Genov KA, Komaroff E, Li Y, Lin L (2006). Nickel-titanium rotary instrument fracture: a clinical practice assessment. Int Endod J.

[13] Parashos P, Messer HH (2006). Rotary NiTi instrument fracture and its consequences. J Endod.

[14] Grande NM, Plotino G, Pecci R, Bedini R, Malaquino VA, Somma F (2006). Cyclic fatigue resistance and three-dimensional analysis of instruments from two nickel-titanium rotarysystems. Int Endod J.

[15] Inan U, Aydin C, Tunca YM (2007). Cyclic fatigue of ProTaper rotary nickel-titanium instruments in artificial canals with 2 different radii of curvature. Oral Surg Oral Med OralPathol Oral Radiol Endod.

[16] Peng B, Shen Y, Cheung GS, Xia TJ (2005). Defects in ProTaper S1 instruments after clinical use: longitudinal examination. Int Endod J.

[17] Zelada G, Varela P, Martín B, Bahíllo JG, Magán F, Ahn S (2002). The effect of rotational speed and the curvature of root canals on the breakage of rotary endodonticinstruments. J Endod.

[18] Patiño PV, Biedma BM, Liébana CR, Cantatore G, Bahillo JG (2005). The influence of manual glide path on the separation rate of NiTi rotary instruments. J Endod.

[19] Booth JR, Scheetz JP, Lemons JE, Eleazer PD (2003). A comparison of torque required to fracture three different nickel-titanium rotary instruments around curves of the sameangle but of different radius when bound at the tip. J Endod.

[20] Iqbal MK, Kohli MR, Kim JS (2006). A retrospective clinical study of incidence of root canal instrument separation in an endodontics graduate program: a PennEndo databasestudy. J Endod.

[21] Vieira EP, França EC, Martins RC, Buono VT, Bahia MG (2008). Influence of multiple clinical use on fatigue resistance of ProTaper rotary nickel-titanium instruments. Int Endod J.

